# Supplementary material for: Mapping the evolution of fertility support policies in China: A content and instrumental analysis
Source: PLoS One. 2025 Oct 9;20(10):e0332137. doi: 10.1371/journal.pone.0332137 (PMC12510515; doi:10.1371/journal.pone.0332137)
Supplement: S1 Appendix — (ZIP) [file pone.0332137.s001.zip › S1 Appendix. 226 original policy documents/216-第七届全国人民代表大会第三次会议关于1989年国民经济和社会发展计划的执行情况、1990年国民经济和社会发展计划的决议(FBM-CLI-1-175084).doc]

第七届全国人民代表大会第三次会议关于1989年国民经济和社会发展计划的执行情况、1990年国民经济和社会发展计划的决议

**发布部门：** [全国人民代表大会](https://www.pkulaw.com/law/chl?Aggs.IssueDepartment=101)

**发布日期：**1990.04.04

**实施日期：**1990.04.04

**时效性：** [现行有效](https://www.pkulaw.com/law/chl?Aggs.TimelinessDic=01)

**效力级别：** [工作文件](https://www.pkulaw.com/law/chl?Aggs.EffectivenessDic=XA0105)

**法规类别：** [人大议事](https://www.pkulaw.com/law/chl?Aggs.Category=00106)

第七届全国人民代表大会第三次会议关于1989年国民经济和社会发展计划的执行情况、1990年国民经济和社会发展计划的决议
（1990年4月4日第七届全国人民代表大会第三次会议通过）

　　第七届全国人民代表大会第三次会议经过审议并根据全国人民代表大会财政经济委员会的审查报告，决定批准国务院提出的1990年国民经济和社会发展计划，批准国务委员兼国家计划委员会主任邹家华所作的《关于1989年国民经济和社会发展计划执行情况与1990年计划草案的报告》。

关于1989年国民经济和社会发展计划执行情况与1990年计划草案的报告
--1990年3月21日在第七届全国人民代表大会第三次会议上
国务委员兼国家计划委员会主任 邹家华

　　现在，我受国务院委托，向大会提出1989年国民经济和社会发展计划执行结果与1990年计划安排情况的报告，请予审议。
　　一、1989年计划的执行情况
　　1989年，在中国共产党和人民政府领导下，经过全国各族人民的团结奋斗，我们取得了制止动乱和平息暴乱的历史性胜利，在国际风云变幻中继续推进了各项社会主义建设事业。整个计划的执行情况比较好，国民经济在治理整顿和深化改革中正朝着好的方向发展。突出表现在：连续几年的经济过热已经降温，过大的固定资产投资规模有了压缩，通货膨胀得到控制，全国零售物价上涨幅度逐月降低，社会生产继续增长。主要成绩有以下几个方面：
　　第一，粮食生产获得好收成，农林牧副渔业继续发展。全国农业总产值达到6550亿元，按可比价格计算，比上年增长3.3%。尽管去年部分地区遭受了较为严重的自然灾害，但由于广大农民的努力和各方面的支援，以及增加了种植面积，粮食生产仍比上年有较多增长，总产量4.0745亿吨，达到历史最好水平。与人民基本生活需要关系密切的蔬菜、水果、肉类和水产品，都有较大幅度的增产。由于播种面积减少，棉花、油料和糖料产量比上年有所下降。农业生产的稳定增长，是全国经济比较稳定的重要条件。
　　第二，连续几年过高的工业增长速度已经降了下来。全国工业总产值达到21880亿元，按可比价格计算，比上年增长8.3%（不包括村及村以下工业为6.8%），达到了计划的要求。其中，轻工业总产值增长8.4%；重工业总产值增长8.2%。工业生产结构有所改善。能源生产总量和重要原材料的生产稳步增长。原煤产量突破10亿吨，达到10.4亿吨，比上年增长6.1%；发电量达到5820亿千瓦小时，增长6.7%；钢产量达到6124万吨，钢材产量达到4865万吨，分别增长3%和3.7%。化肥、农药等支农产品增长较快。机电工业和轻纺工业在调整产品结构和企业组织结构方面开始取得进展，前几年增长过快的一般加工工业有较大回落，特别是家用电器等耐用消费品的生产下降幅度较大。交通运输部门通过挖掘潜力、提高运输效率，货物周转量比上年增长7.2%。邮电通讯事业有较快发展。
　　第三，固定资产投资规模得到控制，一批大中型项目建成投产。由于各部门、各地区认真抓了清理在建项目和压缩投资规模的工作，1989年全社会固定资产投资完成4000亿元，比上年减少近500亿元，下降11%，扣除价格因素，实际工作量压缩20%以上。全民所有制单位基本建设和更新改造施工项目12.3万个，比上年减少4.3万个；当年新开工项目3.7万个，比上年减少4.1万个。在连续多年投资膨胀的势头得到抑制的同时，投资结构有所调整。全民所有制单位能源工业部门和运输邮电部门的投资比重，都比上年提高。生产性建设投资所占的比重，由上年的65.9%上升到68.4%；非生产性建设投资的比重，由34.1%下降到31.6%，特别是楼堂馆所的建设得到严格控制。重点建设有所加强。全国建成投产大中型基本建设项目57个；大中型项目的单项工程128个。新增原煤开采能力2495万吨，原油开采能力1705万吨，发电装机容量902万千瓦，铁路复线318公里，沿海港口吞吐能力4885万吨。现有企业技术改造取得新的成绩。全国建成投产2.75万多个技术改造项目，扩大了一些重要短缺产品的生产能力，提高了企业的生产技术水平。所有这些，都为经济发展增加了后续能力。
　　第四，市场商品供应比较充裕，零售物价上涨幅度低于上年。社会商品零售总额为8101亿元，比上年增长8.9%。吃、穿、用商品和农业生产资料的销售额继续增长。棉布、自行车、缝纫机、家用电器等商品销量在前几年大幅度增长的基础上，有不同程度回落。1989年全国零售物价总水平比上年上涨17.8%，上涨幅度低于上年，特别是从10月份开始物价上涨幅度已降到两位数以下。人民基本生活必需品的价格基本稳定，大体保持在上年12月份的水平。一些商品价格稳中有降。
　　第五，进出口贸易持续增长，对外经济技术交流继续发展。据海关统计，全年进出口货物总额达到1116亿美元，比上年增长8.6%。其中，出口总额525亿美元，增长10.5%；进口总额591亿美元，增长7%。扣除来料加工、外资企业作为投资进口的设备等不收付外汇的货物后，进出口逆差为28.5亿美元。非贸易现汇收大于支30亿美元。全年实际利用外资继续增长。国际收支情况有了改善。经济特区和沿海开放地区外向型经济进一步发展。
　　第六，科技、教育和文化、卫生、体育等各项事业稳定发展。1989年，科技战线取得国家自然科学奖60项，国家发明奖150项，科技进步奖504项，“星火”奖123项。一些领域的科技成果接近或达到国际先进水平。国家重点科技攻关取得了一批重大成果，高技术计划实施进展顺利。重点企业的技术开发和科学技术成果推广取得新进展，技术市场更加活跃。基础性科学研究进一步加强。教育事业在调整结构中向前发展。基础教育继续加强，全国通过普及初等教育检查验收的县达到1389个，比上年增加63个。中等职业技术教育稳步发展。成人技术培训学校在校学生大幅度增长。普通高等学校招生规模有所控制，结构有所改进。文学、艺术、广播、电影、电视、新闻、出版、文物等各项文化事业都有新的发展。城乡居民的卫生医疗条件进一步改善。体育事业取得好成绩。
　　第七，社会消费需求有所控制。据国家统计局抽样调查，1989年城镇居民平均每人可用于生活费的收入为1260元，比上年增长12.6%；农民平均每人纯收入602元，比上年增长10.5%。扣除价格变动因素，一部分城乡居民的实际收入略有下降。社会集团消费得到有效控制。城乡居民储蓄大幅度增加，1989年末居民储蓄存款余额达到5135亿元，比上年末增加1334亿元，增长35.1%。增长幅度这么大，是多年来所没有的。城乡居住条件继续改善，城镇新建住宅1.6亿平方米，农村新建住宅7.1亿平方米。社会福利事业和保险事业进一步发展。
　　总的来看，1989年国民经济和社会发展计划执行情况是好的，这是各地区、各部门坚决贯彻执行治理整顿和深化改革的方针，广大干部和群众团结一致、克服困难、辛勤劳动的结果。我代表国务院向为我国经济和社会稳定发展做出贡献的广大工人、农民、知识分子、人民解放军指战员、干部和各界人士，致以诚挚的敬意和感谢。
　　尽管治理整顿工作取得了比较明显的成效，但是经济生活中还存在着不少问题和困难。主要是：（一）多年积累下来的国民收入超分配、社会总需求超过总供给的问题，还没有得到根本解决。庞大的社会结存购买力，依然是市场稳定的严重威胁。（二）去年第四季度以来，市场销售疲软，资金紧张，产成品库存积压，商品流通不畅，工业生产的增长速度回落过猛。（三）结构调整进展缓慢。一些长线的、质次价高的产品仍在增产，而一些紧缺的、适销对路的产品则增长不快。基础产业的重点建设资金不足，不能适应国民经济发展的需要。加工工业的行业改组和企业组织结构调整，同治理整顿的要求还有相当大差距。（四）经济效益差的状况仍然没有改变。1989年预算内国营工业企业实现利税，仅比上年增长0.2%；定额流动资金周转天数，由上年的97天延长到108天，产成品积压严重；产品成本上升，企业亏损大幅度增加。（五）停产、半停产企业增加，城镇待业人员增多，部分居民生活发生困难。这些问题，不少是原有矛盾的进一步暴露，有些是治理整顿中难以避免的，有的则是在新情况下出现的新矛盾。从我们工作上说，也存在一些缺点，主要是在紧缩需求的操作过程中，由于缺乏经验，在宏观经济疏导方面采取的措施不够及时有力，注意适时适度调节不够。总之，影响经济稳定发展的一些深层次问题还没有从根本上得到解决，导致通货膨胀的一些基本因素还没有消除，新老矛盾交织在一起，治理整顿的任务还很艰巨。我们一定要肯定成绩，正视困难，看到经济发展的有利条件和光明前景，增强信心，总结经验，改进工作，坚定不移地把治理整顿和深化改革继续推向前进。

　　二、1990年计划的主要目标和任务
　　1990年，是九十年代的第一年，也是治理整顿和深化改革的关键一年。安排好今年的计划，对于做好全年的经济工作，逐步实现治理整顿的任务和促进九十年代国民经济持续稳定协调发展，都具有重要的意义。按照中国共产党十三届四中、五中、六中全会精神，1990年计划安排的指导思想是，坚定不移地贯彻执行党在社会主义初级阶段的基本路线，坚持以经济建设为中心，坚持四项基本原则，坚持改革开放，调动一切积极因素，团结全国各族人民，振奋精神，克服困难，继续推进治理整顿和深化改革，为我国政治、经济和社会的进一步稳定发展而奋斗。在巩固和发展安定团结政治局面的前提下，集中精力把国民经济搞上去。今年的治理整顿和深化改革，要充分依靠广大人民群众，在继续坚持和改进经济总量控制的同时，着重调整经济结构，加强各项管理，发展科学技术，提高人员素质，增进经济效益，保证国民经济以适当速度持续增长。各项计划安排中，要真正体现过几年紧日子的要求，并注意统筹兼顾、瞻前顾后。把治理整顿和深化改革更加紧密地结合起来，在保持基本政策稳定性、连续性的基础上，进一步深化和完善各项改革措施，使它们有利于和服务于治理整顿。
　　基于上述指导思想，1990年国民经济和社会发展计划的主要目标是：
　　--保持经济适度增长率。在提高经济效益和技术水平的基础上，农业总产值比上年增长4％；工业总产值增长6%；国民生产总值增长5%。
　　--进一步降低通货膨胀率，全国零售物价上涨幅度计划控制在低于上年水平。
　　--全社会固定资产投资规模计划4100亿元，并切实按照国家产业政策，进一步调整投资结构。
　　--继续从紧控制信贷规模和货币发行量，国家预算赤字低于上年水平。
　　--保持当年外汇收支基本平衡。
　　1990年计划的主要任务，有以下几个方面：
　　（一）切实加强农业，争取粮食，棉花等主要农产品生产稳定增长。
　　今年经济工作的一件大事，是齐心合力把农业搞上去，为粮食、棉花生产再上一个新台阶迈出坚实的一步。计划安排粮食产量4.125亿吨，比上年增加504万吨；棉花产量440万吨，增加61万吨；油料作物产量1500万吨，增加209万吨；糖料作物产量6400万吨，增加607万吨。同时，争取其他农产品也有较好收成。继续发展肉、禽、蛋、奶和水产品的生产。肉类总产量安排2550万吨，比上年增加40万吨。
　　实现农业生产稳定增长的关键，是认真落实中央确定的加强农业的各项政策措施。各级政府都要高度重视农业，进一步加强对发展农业的领导。要继续深化农村改革，稳定和进一步完善家庭联产承包责任制，健全统分结合的农村双层经营体制，积极发展各种社会服务体系；在有条件的地方，根据农民自愿的原则，稳步发展适度规模经营和新的集体经济。努力增加对农业的投入。在今年中央掌握的基本建设投资没有增加的情况下，中央对农业的投资计划增加10亿元，比上年增长近30%。中央增加的投资，重点用于大江大河的治理，扩大灌溉面积，建设粮、棉等重要商品生产基地等。省地市县也要把地方财力优先用于发展农业生产，增加农业投资。银行和信用社要积极组织好农用资金的筹集和投放。要把各种渠道用于农业的资金统筹安排、管好用好。同时，进一步采取有力政策，引导农村集体经济和农民把更多的资金投入农业生产和开发，增加劳动积累。积极增加化肥、农药、农膜等农业生产资料的生产和供应。在全国成品油供应量减少的情况下，农用柴油仍维持去年计划水平。继续完善重要农业生产资料的专营工作，并努力保持价格基本稳定。为了保证粮、棉稳定增产，今年国家下达了分地区的粮、棉播种面积指导性计划，全国粮食播种面积力争达到16.83亿亩、棉花播种面积8350万亩，分别比上年增加600万亩和500万亩。大力推广一批花钱较少、增产显著的实用农业科技成果，并适当增加鼓励农业科学研究、支持农业技术开发和推广的经费。国务院决定，今年将提高棉花、油料以及糖料、烤烟的收购价格，以协调农产品内部的比价关系，进一步调动农民对各类农产品的生产积极性。坚决制止乱摊派、乱收费，减轻农民负担。
　　（二）积极促进市场销售，大力调整结构和提高经济效益，保持工业生产适度增长。
　　努力实现工业生产达到计划增长6%的速度，对于增加有效供给、缓解财政困难、稳定经济全局，都有着重要意义。计划安排，原煤产量10.6亿吨，比上年增加2000万吨；原油产量1.38亿吨，增加35万吨；发电量6050亿度，增加230亿度；钢产量6000万吨到6100万吨；纱产量2300万件；化学纤维产量147万吨；铁路货运量14.7亿吨。
　　近一个时期来，由于市场销售疲软等原因，工业生产处于低速增长状态。市场销售所以疲软，深层的原因是多年造成的结构不合理，经济效益差。因此，要扭转这种状况，使工业生产以适当速度增长，必须把工作的重点放在调整结构和提高经济效益上。
　　各地区、各部门都应当采取切实有力的措施，抓紧结构调整。要努力增加能源、原材料短线产品的生产。煤炭工业要继续抓好国家直属煤矿生产，同时积极发展地方煤矿生产。特别是河北、河南、安徽、山东、辽宁、黑龙江等地，要尽量多生产一些煤炭，以缓解煤炭运输的压力。电力工业，要组织好火电和水电的生产，努力降低厂用电和线路损失，保证电力质量，进行合理分配和调度，以尽可能满足用户特别是重点用户的需要。原材料工业，特别是钢铁工业要努力增加矿石生产，着力于提高产品质量，增加短线品种。轻纺工业，要大力调整产品结构，压缩和控制长线产品、质次价高产品，开发和增加在国际市场和国内市场上适销对路的新产品、新品种、新款式，特别是要抓好人民生活必需的日用工业品和小商品生产。机电工业，要控制和压缩高耗能、低水平的产品生产，积极开发为基础产业服务的技术装备和节约能源、原材料的新产品，加强基础元器件、基础机械、基础工艺的攻关和生产，提高产品性能和成套装备水平，努力发展出口创汇产品和进口替代产品。交通运输部门，要进一步挖掘潜力，提高运输系统的综合效率。为了保证能源、运力的稳定增长，今年要适当提高原油和铁路、水运的货运价格。
　　所有工业部门和生产单位，必须真正做到以提高经济效益为中心，大力提高产品质量，降低物质和能源消耗，从提高效益中求生存、求稳定、求发展。为此，一要加强企业的技术改造，积极推广降低消耗、提高质量和投入少、产出多的科技成果，加速对引进技术的消化、吸收和创新，发动群众开展小改小革，努力提高企业的生产技术水平。二要强化企业管理，从基础工作抓起，建立健全并严格执行各种工艺规程、劳动纪律和规章制度，努力提高各方面的管理水平。三要普遍深入开展增产节约、增收节支运动，反对各种铺张浪费，精打细算，厉行节约，大力扭亏增盈。计划要求，全年直接和间接节约能源1000万吨到1500万吨标准煤，全民所有制工业全员劳动生产率提高3%。各地区、各部门都要制定提高经济效益的明确目标和具体措施，并层层加以落实。
　　为了改变市场销售疲软、工业生产低速增长的状况，还要在坚持财政、信贷双紧方针的前提下，从多方面采取一些缓解矛盾的疏导措施。要适当增加用于工业生产和商业、外贸、物资收购的贷款。适当调整存、贷款利率，并在贷款上实行差别利率。尽快清理企业之间的“三角债”，全面恢复银行托收承付的结算方式。按计划拨足国家重点和急需工程所需的建设资金，同时适当增加技术改造贷款。进一步搞活流通，大力开拓市场特别是农村市场，努力增加出口。上述这些措施的贯彻实施，将会促进工业生产以适当的速度稳定增长。
　　（三）继续控制固定资产投资规模，调整投资结构。
　　1990年计划安排，全社会固定资产投资规模4100亿元。其中，全民所有制单位固定资产投资为2510亿元，集体所有制单位和城乡个体投资为1590亿元。在全民所有制单位固定资产投资中，基本建设投资为1350亿元；更新改造投资为820亿元。
　　在控制基本建设投资规模的前提下，计划对投资结构作了调整。主要是压缩一般性建设投资，重点加强农业、能源、交通等方面的投资。在今年的计划安排中，由于国家投资有限，过去铺开的建设战线过长，所以有些重点行业最低需要的投资还不能完全得到保证，有些项目要推迟进度。这对增加经济发展的后续能力会有一定影响，但为了控制投资规模，稳定经济全局，也只能作这样安排。在计划执行中，就全国范围来说，要把投资规模控制在不超过上年实际工作量的水平，并优先保证在建重点项目的需要，努力使它们按照合理工期的要求及时建成投产。
　　除了必要的新建以外，要把更多的注意力放在对现有企业技术改造上。更新改造投资的安排，重点是节约能源、原材料，提高产品质量、增加短线品种和新品种的项目，以及扩大出口创汇和进口替代的项目，努力促进产品升级换代。更新改造资金必须真正用于技术改造，不能拿去搞基本建设。对于今年能见成效的技术改造和设备更新项目，要在资金上给予优先保证。同时，企业的技术改造必须促进产品结构的调整和企业组织结构的合理化。
　　（四）安排好国内市场和人民生活，继续控制物价总水平。
　　稳定市场、稳定物价，是稳定经济、安定人民生活的重要方面。1990年计划安排，社会商品零售总额9300亿元，比上年增长14.8%。为了稳定国内市场，一方面要继续控制消费需求的过快增长，加强工资基金计划管理和工资奖金以外收入的管理，加强和改进对城乡集体和其他经济成份个人收入的管理和调节，继续控制社会集团购买力；另一方面，要努力增加有效供给。各地区要继续抓好“菜篮子”，组织好大中城市肉、蛋、菜的生产和供应，安排好城乡居民所必需的日用工业品的生产和供应。要充分发挥国营商业和供销社的主渠道和“蓄水池”作用，抓好农副产品的收购，做好主要商品的购销的地区间的调剂工作。要采取多种有效措施扩大销售渠道，改进商业服务质量，增加商品性货币回笼。
　　今年既要继续控制物价上涨总水平，又要适当调整某些产品价格，以扶持和增加基础产品的生产。在这种情况下，要把物价上涨控制在计划要求的幅度内，任务相当艰巨，必须采取综合配套措施。一是有效地控制社会总需求，这仍然是控制物价上涨幅度的根本性措施。二是各生产、流通、建设单位都要努力提高经济效益，消化一部分涨价因素。三是继续抓好清理整顿公司工作，坚决取消重利盘剥、哄抬物价、扰乱市场的中间环节，严禁非法倒买倒卖。四是整顿市场秩序，加强对重要商品价格的管理，适当集中价格审批权限，认真执行商品和劳务收费的明码标价制度，继续实行控制物价的目标责任制。五是努力稳定人民基本生活必需品的价格和劳务收费，有的要明确规定不准涨价。为了有效地控制物价上涨幅度，对计划确定的调价措施，要精心组织实施，认真做好工作。
　　由于降低工业速度、调整经济结构和压缩投资规模，一些企业停产半停产，要稳妥安排好这些企业人员的工作和生活，组织他们从事多种有益活动，不要把他们推到社会上去。要区别不同情况，发给适当的工资或生活费，保障他们的基本生活。今年城镇新成长的劳动力有几百万人，加上原有的待业人员，今年安置待业人员的任务相当繁重。计划要求，各地区、各部门要积极开拓就业门路，尽量减少待业人员，同时要健全和完善待业保险制度，帮助待业人员克服暂时困难。
　　（五）坚持对外开放，努力调整进出口商品结构，进一步发展对外贸易和经济技术交流。
　　坚持对外开放，更有成效地扩展对外贸易和经济技术交流，既是推进我国现代化建设必须长期坚持的战略方针，又是克服当前经济困难、搞好治理整顿的重要措施。要充分利用国际上的有利条件，克服不利因素，认真做好各项工作，使对外开放和治理整顿更好地互相促进。
　　努力扩大出口。计划安排，外贸出口比上年计划略有增加，力争在执行中超过。除了组织好传统的、大宗的出口商品货源并尽量增加出口外，要根据国内压缩需求和调整结构以后的市场情况，积极组织那些供需矛盾缓和的商品和原材料的出口，特别要努力扩大机电产品等工业制成品以及深度加工和高技术产品的出口，继续增加轻纺产品和创汇农产品的出口。对扩大出口所必需的资金、外汇和原材料、运力，要尽力给予支持。要努力提高出口产品的质量，改进出口贸易的管理。在扩大出口的同时，合理安排进口。对那些国内能够生产供应的原材料和机电设备，要少进口或不进口，严格限制奢侈品和高档消费品的进口。地方外汇首先要用于生产建设和人民生活急需的物资和商品的进口。切实加强和改进进口贸易的管理，对限制进口和实行配额进口的商品，要严格执行国家规定，任何部门、地方和单位不得违反。
　　继续抓好利用外资和引进先进技术的工作。努力帮助办好一批已建成的中外合资、合作企业，以增强对外商投资的吸引力。要把吸收外商直接投资作为利用外资的重点。加强外商投资的引导工作，使之更好地符合我国的产业政策。切实加强对外债借、用、还三个环节的管理。进一步办好经济特区和沿海开放城市的经济技术开发区，鼓励和支持沿海地区发展外向型经济。
　　（六）进一步发展科学、教育和文化卫生体育事业，严格控制人口增长。
　　科学技术是第一生产力。要显著提高社会经济效益，实现经济持续、稳定、协调发展，必须把促进科学技术进步放到重要战略地位。今年的科学技术工作，要把“科技兴农”和推动工交企业技术进步作为中心任务。继续认真抓好“七五”期间国家重点科技攻关计划的实施，按照计划确定的目标和进度，努力完成20项重大成套技术、100项新技术新工艺、400项重点新产品的攻关任务。抓紧抓好国家高技术计划、重点企业技术开发计划的落实工作。要重视大型研究院、所、高等院校和大中型企业的骨干作用，把它们的科技力量更好地组织起来，协同配合，完成国家紧迫的科技任务。同时，要把近期科技进步同中长期科技发展很好结合起来。
　　继续办好各级各类学校。所有学校都要坚持正确的政治方向，切实加强思想政治教育，真正把德育放在首位，促进学生德、智、体全面发展。高等学校要继续控制发展规模，积极调整结构，提高教育质量。计划安排，研究生招生3万人，维持上年水平；普通高校招生62万人，比上年增加2.3万人，其中包括从农业、林业和教育等战线试招一部分有实践经验的在职人员。对达不到设置标准的学校要进行整顿和调整。继续加强中等职业教育和基础教育，努力提高成人教育水平。采取各种措施，不断改善基础教育的办学条件，努力制止中小学生辍学。要加强对社会各类人员的职业道德教育。为了确保教育事业在治理整顿中稳定发展，今年在财政相当困难的情况下，国家继续增加了用于教育的资金。各级政府还要调动社会各方面的积极性，努力使办学条件得到改善。
　　文学艺术、广播、电影、电视、新闻、出版、文物、档案等各项文化事业，要坚持为社会主义、为人民服务的方向，贯彻百花齐放、百家争鸣的方针，坚决反对资产阶级自由化，弘扬民族优秀文化，努力为人民提供更多、更好的精神食粮，促进社会主义精神文明建设，在稳定社会和鼓舞人民方面发挥积极作用。
　　卫生事业，重点要加强预防保健和农村卫生工作。继续增加医院病床，努力改进医疗服务质量。体育事业，要在广泛开展群众性体育活动、增强人民体质的同时，积极准备和组织好第十一届亚运会，鼓励广大运动员刻苦锻炼，努力提高竞技水平，争取在亚运会和其他国际比赛中取得好成绩。
　　控制人口增长、提高人口素质，是关系我国社会主义现代化事业的大事。各级政府要切实加强领导，坚持不懈地抓好计划生育工作。坚决稳定现行的计划生育政策，并使之在基层真正得到落实。要进一步采取有力措施，包括积极推行计划生育指标管理，切实控制人口增长，重点要把农村过高的出生率降下来。各级政府对计划生育所需经费，要尽量给予支持。

　　三、坚持把治理整顿和深化改革结合起来，努力完成1990年计划
　　1990年国民经济和社会发展计划的任务是相当艰巨的。为了顺利实现计划确定的各项任务，必须按照党的十三届四中、五中、六中全会精神进一步统一思想，正确处理治理整顿和深化改革的关系，使它们紧密结合，互相促进。按照中央确定的治理整顿期间深化和完善改革的要求，根据计划经济与市场调节相结合的原则，稳定、充实、调整和改善前几年的改革措施，并在继续搞活微观经济的同时，适当加强集中，积极改进和加强宏观调控体系的建设。
　　第一，坚持和完善企业承包经营责任制。今年要保持经济的适度增长，在调整结构、提高效益方面取得明显进步，关键在于深化企业改革，充分调动企业和职工的积极性。这几年工商企业实行各种形式的承包经营责任制，总的说效果是好的，要继续保持稳定，并认真总结经验，兴利除弊，不断完善和发展，特别要注意克服短期行为的倾向，培育企业自觉进行技术改造、推动技术进步的机制。同时，深化企业内部改革，健全各项规章制度和责任制。坚持和完善厂长负责制，充分发挥企业党组织的政治思想领导作用。继续发展企业集团，推动现有生产要素的优化组合和企业组织结构的调整。
　　大中型国营企业是我国国民经济的重要基础和骨干，要充分发挥它们的应有作用。今年国家在资金、物资和运力的分配上将实行重点倾斜措施，优先保证那些产品适销对路、经济效益好的大中型企业的需要。同时，对那些符合产业政策、经济效益和社会效益好的中小企业，实行适当的保护政策。对城乡集体企业和乡镇企业，要按照调整、整顿、改造、提高的方针，积极加以引导，促进它们健康发展。
　　第二，加强对生产、流通领域的计划指导和管理。根据治理整顿和深化改革的要求，适当调整指令性计划和指导性计划的范围。在工业生产方面，今年增加了一些重要原材料和稀缺产品的指令性计划指标。农业生产方面，恢复了一些重要产品播种面积的指导性计划。同时，要制定改进指令性计划和指导性计划的实施办法。适当增加某些重要和紧缺的物资由国家统一分配的部分；对企业自销的某些重要生产资料采取划出一块，由国家导向销售的办法，以利于保证国家重点生产和建设的需要。进一步整顿流通环节和市场秩序。今年开始对国家直属煤矿生产的煤炭、地方上交国家的部分煤炭以及经铁路运输的计划外出省煤炭，实行统一分配，统一订货，统一调度，统一运输。除了国家批准和有关部门指定的经营单位以外，其他单位和个人一律不得经营煤炭。对计划外自销的重要生产资料实行公开销售制度和最高限价，有关部门要抓紧制定具体实施办法。要加强对地区协作的计划指导。
　　第三，继续深化固定资产投资体制的改革。为了有效控制固定资产投资规模和调整投资结构，从今年起，国家对基本建设实行年度投资规模和在建项目投资总规模双重控制的办法。继续适当集中新开工项目的审批权。大中型基本建设项目和限额以上的更新改造项目，仍由中央审批；属于国家限制发展产业的建设项目，即使投资低于限额的，也应集中审批权。属于地方的审批权限，要适当集中到省一级，并克服审批权多头分散的现象。开征投资方向调节税（同时取消建筑税），按国家产业政策实行差别税率，把一部分非重点建设投资转到国家重点建设上来。要进一步建立和完善投入和产出挂钩的投资包干责任制，更好地推行招标投标制度，逐步克服“吃大锅饭”的弊端。同时，整顿建设领域秩序，努力提高投资效益。今年，要对一批大中型基本建设项目进行审计检查。
　　第四，按照计划经济与市场调节相结合的原则，加强和改进宏观调控体系和制度。进一步制定和完善国家产业政策的实施办法和方案，并组织有关部门和地区通力协作，努力落实各项配套措施，并加强监督和检查，使国家产业政策真正落到实处。财政包干体制今年继续实行，但要按照适当集中财力的原则，区别不同情况，适当提高地方上交中央财政的数额，适当减少中央对地方的补贴。同时，积极进行分税制的试点。进一步强化中央银行的宏观调控职能，加强对专业银行的归口领导和管理。中央银行要加强资金管理，搞好适时调节。各专业银行要严格执行国家产业政策和国家计划，不能片面强调企业化经营。正确发挥利率杠杆对资金需求和资金流向的调节作用。积极探索以国家计划为主要依据的经济、行政、法律手段综合运用的宏观调控体系，抓紧基本经济法律、法规的制定工作。
　　各位代表！完成今年国民经济和社会发展计划的任务是艰巨而又光荣的。尽管当前我国经济遇到一些暂时困难，但是也有许多克服困难的有利条件。经过10年来的建设和改革，我国经济实力有了很大增强，人民生活有了显著改善；经过一年多来的治理整顿，我们已经取得了比较明显的成效，积累了不少经验；党的十三届四中、五中、六中全会的召开，全党的思想认识更加统一，团结更加增强。只要我们坚定不移地贯彻以经济建设为中心、坚持四项基本原则、坚持改革开放的基本路线，坚决依靠和紧密团结全国各族人民，发扬光荣传统，振奋精神，埋头苦干，自力更生，艰苦奋斗，就一定能够全面完成1990年计划，逐步实现治理整顿和深化改革的预期目标，促进政治、经济和社会的进一步稳定和发展。
　　相关文件: 1. 第七届全国人民代表大会财政经济委员会关于1989年国民经济和社会发展计划执行情况、1990年国民经济和社会发展计划草案的审查报告 2. 第七届全国人民代表大会第三次会议关于1989年国民经济和社会发展计划的执行情况、1990年国民经济和社会发展计划的决议 3. 关于1990年国民经济和社会发展计划执行情况的汇报

第七届全国人民代表大会财政经济委员会关于1989年国民经济和
社会发展计划执行情况、1990年国民经济和社会发展计划草案的审查报告
（1990年3月30日第七届全国人民代表大会第三次会议主席团第二次会议通过）
全国人大常委会副委员长、全国人大财政经济委员会主任委员 陈慕华

　　第七届全国人民代表大会第三次会议，于1990年3月21日听取了国务委员兼国家计划委员会主任邹家华所作的《关于1989年国民经济和社会发展计划执行情况与1990年计划草案的报告》，各代表团进行了审议。全国人民代表大会财政经济委员会根据代表们审议的意见，对报告进行了讨论。现将审查意见报告如下：

　　一、1989年是很不寻常的一年。第七届全国人民代表大会第二次会议刚刚审议批准1989年国民经济和社会发展计划，全国上下正在为实现计划努力奋斗的时候，极少数人利用学潮，掀起了一场政治动乱，进而在北京发展成为反革命暴乱，严重地妨碍了计划的实施，给国民经济造成了相当大的损失。面对这种严峻的形势，全国各族人民在党和政府的领导下，平息了动乱和暴乱，努力生产，勤奋工作，使我国经济在很困难的条件下仍然取得了比较好的成绩，国民经济和社会发展计划的执行情况基本上是好的。主要表现在：社会总需求与总供给的矛盾趋于缓和，粮食生产获得较好收成，过高的工业增长速度明显回落，固定资产投资有所压缩，消费需求增长减缓，货币发行明显减少，物价涨势得到控制，进出口贸易继续发展，外汇储备有了增加。国民经济正朝着好的方向发展。
　　1989年计划执行中也还存在一些问题，棉花、油料、糖料的种植面积减少，产量下降；压缩固定资产投资规模没有达到计划要求；企业成本上升，经济效益继续下降；物价上涨幅度没有达到明显低于上年的计划要求；财政赤字突破了预算。同时，在治理整顿中又出现了一些新的问题，主要是去年第四季度以来市场销售不旺，工业生产速度回落过猛，产成品积压，流动资金短缺，停产半停产企业增多，部分职工生活水平下降。这些问题的出现，是多年积累下来的一些深层次矛盾的反映，是治理整顿过程中难以避免的；有的则是由于工作缺乏预见性，措施不够及时，调度不够有力所致。我们要肯定成绩，正视困难，总结经验，改进工作，坚定不移地把治理整顿和深化改革继续推向前进。

　　二、国务院提出的1990年国民经济和社会发展计划草案，体现了中国共产党十三届四中、五中、六中全会的精神，继续推进治理整顿和深化改革，促进我国政治、经济和社会的进一步稳定发展。
　　1990年国民经济和社会发展计划的主要任务是：（一）保持经济适度增长率；（二）进一步降低通货膨胀率，物价上涨幅度要低于上年水平；（三）继续控制全社会固定资产投资规模，进一步调整投资结构；（四）继续从紧控制信贷规模和货币发行量，国家预算赤字低于上年水平；（五）保持当年外汇收支基本平衡。
　　财经委员会认为，国务院提出的计划是可行的。建议全国人民代表大会批准1990年国民经济和社会发展计划，批准国务委员兼国家计划委员会主任邹家华所作的《关于1989年国民经济和社会发展计划执行情况与1990年计划草案的报告》。在执行中，各级政府要及时进行检查，协调和解决执行中的问题，并定期向同级人民代表大会常务委员会汇报计划执行情况。如有重大调整，事先应报同级人民代表大会常务委员会审查、批准。

　　三、为了保证1990年国民经济和社会发展计划的实现，必须统一思想，进一步提高治理整顿和深化改革的自觉性和坚定性。目前，国民经济虽然正在向好的方向发展，但还存在着许多问题和困难。一定要重视并采取措施解决当前经济发展缓慢的新问题，同时也要注意防止需求再次膨胀。要继续坚持“双紧”方针，认真解决多年积累下来的一些深层次的问题，把治理整顿和深化改革继续推向前进，促进国民经济持续、稳定、协调地发展。为此，财经委员会建议：
　　⒈大力发展农业，夺取农业丰收。
　　要认真贯彻以农业为基础的指导思想，加强对农业的领导，确保粮食、棉花等主要农产品稳定增长，继续发展肉禽蛋奶和水产品的生产，促进农林牧副渔全面发展。为此，首先要稳定和进一步完善家庭联产承包责任制；在有条件的地区，要根据农民自愿的原则，稳妥地发展适度规模经营和新的集体经济。要坚决贯彻执行《[土地管理法](https://www.pkulaw.com/chl/bd79a0260b2f3cfdbdfb.html)》和《水法》，切实保护和合理利用土地资源和水资源。二是国家和地方都要尽可能增加对农业的投入，并积极引导农村集体经济和农民把更多的资金、劳力投入农业生产与开发，兴修水利，整治农田，修建道路，改善生产条件。三是积极增加化肥、农药、农膜等农用生产资料的生产，加强和完善农业生产资料的专营工作，搞好供应，保持价格的稳定；要制定政策，鼓励农民多积、多施农家肥。四是逐步建立、健全农村产供销和科技服务体系，大力推广适用的农业科技成果。五是有关部门应迅速采取措施，收购农民手中的余粮和积存的其它农副产品，保护农民的生产积极性。六是坚决制止对农村的各种不合理摊派，切实减轻农民的负担。对于乡镇企业，要按照调整、整顿、改造、提高的方针，加以扶植和引导，适当帮助解决目前的困难，促进其健康发展。
　　⒉继续控制社会总需求仍然是进一步治理整顿的重要任务。
　　今年的固定资产投资规模，特别是全民所有制单位的投资规模必须严格控制在国家计划之内，进一步调整投资结构，重点增加农业、能源、交通等建设和技术改造方面的投资，压缩一般性建设投资，严禁新建楼堂馆所。要继续认真清理固定资产投资在建项目，严格控制新开工项目。新上项目必须经过科学论证、严格审批等建设程序，审批权要适当集中。对违法建设的，要严肃查处。
　　要继续控制消费基金的过快增长。加强对工资基金的管理，改进与完善企业工资同经济效益挂钩的办法，严禁滥发奖金、实物和随意扩大津贴、补贴。对社会集团的消费，要继续严格控制。
　　⒊进一步调整产业结构、产品结构和企业结构，保持工业适度增长，千方百计提高企业的经济效益。
　　各地区、各部门要根据国家的产业政策和经济合理布局的原则，制订具体实施办法，抓紧产业结构的调整，努力增加能源、交通、原材料等基础产业的生产，克服加工工业盲目发展的现象。要抓紧调整产品结构，对质量好、适销对路、出口创汇和替代进口的产品，要优先给予支持，并积极开发新产品、新品种。要大力加强生产调度工作，及时解决生产、流通中的问题，保证工业正常生产。
　　要认真加强企业管理，做好基础工作；同时要通过技术革新和必要的技术改造以及开展群众性的“双增双节”、合理化建议等活动，不断提高产品质量，降低消耗，降低成本，提高劳动生产率，以提高企业经济效益。同时，要采取有力措施切实减轻企业负担。
　　⒋疏通流通渠道，整顿流通秩序，严格控制物价上涨幅度。
　　商业、供销、物资和外贸系统，要积极收购适销对路的产品和优质名牌产品，努力扩大国内、国外两个市场，充分发挥国营商业、供销社和物资系统的主渠道和蓄水池作用。要积极利用集体、个体和私营商业的渠道，促进城乡物资交流；要保护其合法经营和正当权益，严禁投机倒把和掺杂假冒行为。
　　继续清理整顿公司，是整顿经济秩序的一项重要工作，也是廉政建设的重要内容。要坚持抓下去，切实抓出成效，决不能半途而废。
　　继续控制物价，确保物价上涨幅度低于上年水平，努力稳定人民基本生活必需品价格，保障人民生活安定。
　　⒌严格控制信贷规模和货币发行。
　　要按照国家产业政策的要求，调整信贷结构，实行差别利率。信贷规模要控制，但不搞“一刀切”，做到紧中有活，适时调节。对于民族自治地区，要给予适当的照顾和支持。银行、计划、财政部门要在国务院的领导下，采取有力措施清理企业“三角债”，并抓紧研究解决企业流动资金不足的问题。银行要恢复托收承付的结算办法，同时要监督企业清仓利库，减少资金占用。要加强和改进银行工作，在重视银行宏观调控职能的同时，要加强对银行信贷工作的审计与监督。
　　⒍坚持对外开放，发展对外贸易和经济技术交流。
　　要努力扩大出口，合理安排进口，力争外汇收支平衡，略有节余。要有效地利用外资和先进技术，并加快引进技术的国产化进程，增强自力更生的能力。要进一步办好经济特区，鼓励和支持沿海地区发展外向型经济。要采取有效措施，加强对外汇和外债的管理，坚决纠正盲目进口、重复进口和多头举借外债的现象。
　　⒎积极开拓生产和服务门路，妥善安置停产企业职工和城镇待业人员。
　　各级领导要高度重视这一工作，加强领导，统筹安排。企业应组织停工待工人员进行业务、技术培训，检修设备，以及其它公益劳动。要切实保证停工待工人员的基本生活，积极建立社会保险制度。要认真安排好复员和转业军人的工作。要积极开拓生产经营和服务领域，在城镇要支持组织待业人员发展集体、合作经济，广开就业门路；同时，按照国家政策鼓励和引导个体经济和私营经济健康发展，发挥它们在发展生产、搞活流通、增加就业方面的积极作用。
　　⒏进一步发展教育、科学和文化卫生体育事业，提高全民族素质，促进社会主义物质文明和精神文明建设。
　　要坚持教育的社会主义方向，全面加强和改进学校的思想政治工作，加强爱国主义、集体主义和国防教育。继续深化教育改革，调整教育结构，切实提高教育质量和办学效益，促进教育事业更好地为社会主义建设服务。要以加强基础教育，实施九年义务教育为重点，稳定发展各类教育。要进一步贯彻执行尊重知识、尊重人才的方针，改善教师的待遇，稳定教师队伍。继续增加对教育的投入，确保教育事业的优先发展。
　　必须高度重视促进科技进步，把提高经济效益切实转到依靠科技进步和提高劳动者素质上来。财政、税收、信贷部门要对科技事业实行优惠政策。
　　要积极发展有中国特色的社会主义文化事业，继续“扫黄”和除“六害”，在坚持“二为”方针的前提下，努力繁荣创作，做好图书出版发行工作，丰富人民的精神生活，特别要为青少年和儿童多出好书。要支持卫生、体育事业的发展，提高人民健康水平。
　　要坚决贯彻执行计划生育政策，提倡优生优育、晚婚晚育，严格控制人口增长，提高人口素质。要加强对环境保护工作的领导，控制和治理环境污染，保护和改善生活环境与生态环境。
　　⒐把治理整顿与深化改革紧密地结合起来。
　　要进一步深化企业改革，不断完善和发展企业承包经营责任制，克服“以包代管”和其它短期行为，逐步建立企业自我约束和自我推动技术进步的责任制。同时，应继续进行税利分流和税后承包、税后还贷的试点。要认真贯彻《企业法》，坚持和完善厂长负责制，同时充分发挥企业党组织的政治思想领导作用，发挥职工代表大会和工会的作用，全心全意依靠工人阶级办好企业。
　　必须按照计划经济与市场调节相结合的原则，深化经济体制改革，逐步加强和完善国家宏观调控体系。要加强对经济活动的计划指导和管理，进一步改革固定资产投资体制，逐步提高中央财政收入在全部财政收入中的比重，加强中央银行的宏观调控职能。要加快《计划法》、《预算法》、《投资法》、《银行法》、《价格法》、《公司法》、《海商法》等有关宏观管理的立法工作，加强执法情况的监督检查，使经济管理工作逐步走上法制的轨道。
　　1990年是治理整顿和深化改革的关键一年。完成今年的国民经济和社会发展计划，对于我国政治、经济和社会的进一步稳定发展具有重要的意义。我们相信，只要各级政府、各级领导干部认真贯彻党的十三届四中、五中、六中全会精神，坚决执行本次代表大会的决议，坚持党的基本路线，学习雷锋精神，充分调动全国各族人民的积极性，以身作则，自力更生，艰苦奋斗，振奋精神，克服困难，就一定能够完成今年的国家计划。
　　以上意见，请予审议。

© 北大法宝：（[www.pkulaw.com](https://www.pkulaw.com/)) 专业提供法律信息、法学知识和法律软件领域各类解决方案。北大法宝为您提供丰富的参考资料，正式引用法规条文时请与标准文本核对。
欢迎查看所有[产品和服务](https://www.pkulaw.com/helps/2.html)。法宝快讯：[如何快速找到您需要的检索结果？](https://www.pkulaw.com/helps/70.html)[法宝](https://www.pkulaw.com/helps/69.html)V6有何新特色？

扫描二维码阅读原文

**原文链接：**<https://www.pkulaw.com/chl/fb3f4d27f3ea72b3bdfb.html>
